# Process Evaluation of Pragmatic Cluster-Randomized Trials of Digital Adherence Technologies for Tuberculosis Treatment Support: A Mixed-Method Study in Five Countries

**DOI:** 10.3390/tropicalmed10030068

**Published:** 2025-03-06

**Authors:** Norma Madden, Amare W. Tadesse, Chung Lam Leung, Bianca Gonçalves Tasca, Jason Alacapa, Natasha Deyanova, Nontobeko Ndlovu, Nontobeko Mokone, Baraka Onjare, Andrew Mganga, Kristian van Kalmthout, Degu Jerene, Katherine Fielding

**Affiliations:** 1 Division of TB Elimination and Health Systems Innovation, KNCV Tuberculosis Foundation, 2516 AB The Hague, The Netherlands; 2 Infectious Disease Epidemiology, London School of Hygiene and Tropical Medicine, London WC1E 7HT, UK; 3 KNCV Tuberculosis Foundation, Makati City 1227, Philippines; 4 Organization for Appropriate Technologies in Health, 01033 Kyiv, Ukraine; 5 The Aurum Institute, Johannesburg 2194, South Africa; 6 KNCV Tuberculosis Foundation, Dar es Salaam P.O. Box 11013, Tanzania

**Keywords:** tuberculosis, digital adherence technology, treatment support, process evaluation, intervention fidelity, quality of implementation, intervention coverage, intervention reach, digital health

## Abstract

Digital adherence technologies (DATs) could improve the person-centeredness of tuberculosis (TB) treatment. DATs are found to be acceptable, though evidence of their effectiveness is varied. Our objective was to understand the fidelity of DAT interventions within five cluster-randomized trials. Two DATs (smart pillbox, medication labels) were assessed, with real-time adherence data available to healthcare providers (HCPs) on a digital platform in Ethiopia, the Philippines, South Africa, Tanzania, and Ukraine. A framework assessed four components of implementation: inputs (training, support, mobile access), processes (SMS, home visits, platform usage), outputs (DAT engagement, manual dosing), and outcomes (people with TB (PwTB)–HCP relationship). Fidelity was evaluated by quantitative indicators, and content analysis of qualitative sub-studies supplemented some indicators. Engagement with DATs was high among PwTB. Pillbox users showed high levels of sustained engagement (box opening), with digitally recorded doses ranging from 82% to 91%. Differences were observed in login frequency by HCPs to the adherence platform. In Ethiopia, Tanzania, and Ukraine, there was at least one login to the platform on 71% of weekdays per facility compared with the Philippines and South Africa at 42% and 52%, respectively. Intervention fidelity varied among countries, suggesting a need for future work on optimizing implementation.

## 1. Introduction

Tuberculosis (TB) is a leading cause of illness and death globally, affecting about 10 million people each year [1]. Addressing this global challenge requires concerted efforts that improve both treatment coverage and outcomes through person-centered approaches. Lack of adequate treatment support is among the key contributors to poor treatment outcomes [2]. Among other interventions, digital adherence technologies (DATs) have the potential to improve treatment outcomes. However, emerging data from recent studies provided mixed results, suggesting the need for more evidence in this area [3,4,5,6,7,8].

We recently completed cluster-randomized trials in five countries under the Adherence Support Coalition to End TB (ASCENT) project to fill this evidence gap [9,10]. The results from the five trials showed no difference in poor end-of-treatment outcome by trial arm among people with drug-sensitive TB (DS-TB) [11,12]. Findings from accompanying sub-studies, however, showed acceptability of the DATs, suggesting that DATs combined with differentiated care have direct benefits to people with TB (PwTB) [13,14,15,16,17,18]. Part of the reason for the lack of impact in the intervention arm can be explained by the process of implementation and coverage of the interventions.

Several key challenges in the implementation of DATs have been identified previously in low-income and lower-middle income countries, including unreliable internet connections, limited digital literacy, power outages, fear of stigma, and pillbox use during travel [13,19]. In this paper, we provide further data on the process of implementation and coverage of the key components of the interventions with a view to understanding both the context and process of implementing the intervention being studied [20]. Process evaluations provide critical information to enhance understanding of findings from pragmatic randomized controlled trials (RCTs) [21].

Understanding implementation is particularly important for pragmatic trials, as onsite partners rather than research teams deliver the intervention. Both the content of an intervention and how it is delivered can be amended by those applying the intervention; therefore, establishing fidelity to the intervention is key to understanding reasons for success or failure, particularly for complex interventions such as DATs [22]. Factors affecting intervention fidelity exist at individual, local, and national levels, and are important to consider when conducting trials across different settings [23]. When interventions are implemented in different contexts, adaptation is likely to occur. Capturing adaptation can be a function of process evaluations, in addition to understanding the what and how of implementation, mechanism of impact, and context [24]. Our aim was to assess the process, fidelity, and coverage of the interventions under the five pragmatic trials of DATs among adults with DS-TB [9,10].

## 2. Materials and Methods

### 2.1. Study Design and Period

This was a mixed-method process evaluation of the ASCENT trials in the Philippines, Ethiopia, South Africa, Tanzania, and Ukraine from June 2021 to August 2022. The trial protocols are described in detail elsewhere [9,10,25]. In brief, the trials fall closer to the pragmatic side of the pragmatic–explanatory continuum based on PwTB populations, delivery of the intervention, and trial outcomes [26]. Two DAT types, smart pillboxes and medication labels, were evaluated.

Our evaluation framework was based on four components: inputs, processes, outputs, and outcomes (Figure S1, Supplementary Materials). Quantitative indicators focused on the coverage and fidelity of the intervention, complemented by qualitative data. Table 1 summarizes key indicators. Input indicators focused on DAT training and support for healthcare providers (HCPs) and mobile phone access by people with TB (PwTB). Process indicators primarily concentrated on adherence platform data such as treatment days with an automated SMS reminder, percentage of doses manually recorded, and adherence platform logins per facility. Output indicators also focused on platform data for digital dosing and patterns of consecutive manual dosing added >7 days after the scheduled dose day. The outcome indicator was based on quantitative and qualitative sub-study data from PwTB and healthcare providers about whether the intervention improved the PWTB–HCP relationship.

### 2.2. Study Population

The effectiveness-trial participants were adults with DS-TB initiating treatment using a DAT (Table S2, Supplementary Materials). Respondents for associated sub-studies included people with TB using DATs for their DS-TB treatment, HCPs, and other key actors at 10 randomly selected ASCENT facilities in each country.

### 2.3. Description of the Interventions

PwTB using the pillbox received daily audio or audiovisual reminders to take their treatment. For the medication labels, PwTB were expected to send a free SMS to a short code daily when treatment was taken with a code on the medication label. Pillbox opening and receipt of SMS from PwTB, as proxies for doses taken, were captured digitally on the adherence platform. If there was no recording of a dose taken at a country-specific predefined time, an automated SMS reminder to take treatment was sent on the day and the following day in the continued absence of box opening by the PwTB or SMS. No reminders were sent in Ukraine based on the decision from the Ukrainian research team. In the other four countries, the participant could opt out of the reminder messages that followed when a dose was not confirmed by a set time each day, though information on which participants opted out was not available.

The recording of doses taken on the adherence platform allowed individual-level DAT engagement information to be generated. The TB healthcare provider (HCP) was expected to review these real-time adherence data regularly and initiate differentiated care based on predefined adherence patterns. The adherence platform recorded for each PwTB a treatment day as either: digitally recorded (pillbox opened on the day or SMS sent); manually recorded (no recording that the pillbox was opened or SMS sent, but the HCP confirmed a dose was taken by contacting the PwTB); dose missed (no recording that the pillbox was opened or SMS sent and the HCP confirmed a dose was not taken by calling the PwTB); and unknown (no recording that the pillbox was opened or SMS sent and no additional information from the HCP). HCP confirmation that a dose was or was not taken could take place at any time on or after the treatment-day. The differentiated response algorithm, developed in consultation with stakeholders, was designed to encourage adherence. Actions ranged from educational reinforcements, reminders, and phone calls to home visits.

### 2.4. Context for the Intervention

The infrastructure to deliver the intervention, training of HCPs, and support visits to health facilities made up the resources to support the implementation of the intervention. The infrastructure included the online adherence platform to which both DATs were linked, hosting of the platform in each country, hardware (tablets, desktops), and data services for HCPs to deliver the intervention. The study team focused support on ensuring facility readiness to implement the intervention, including training HCPs on DATs, addressing technical issues, and operationalizing the use of DATs.

### 2.5. Data Collection

TB medication adherence data were available on the platform. Enrollment on either the pillbox or sleeve label was confirmed via the platform, and contact details and treatment start and end dates for PwTB were recorded to operationalize the intervention. A dosing report of each dose day per PwTB using a DAT was recorded and shared by the platform provider.

The adherence platform provider supplied an SMS log for each DAT user. The log contained all SMS sent and received per DAT, including dosing reminders sent to PwTB. A log of support actions taken by HCPs, including phone calls, facility visits by PwTB, or home visits by HCPs, was also available.

Training and implementation support logs were maintained by the research teams in each country. The number of sessions and participants attending DAT training were recorded, along with detailed logs of implementation support visits by study staff to implementing facilities.

Platform usage statistics, including the date, time, duration spent logged in, and number of actions performed per PwTB on a DAT, were recorded automatically on the platform and available by individual HCP login.

For qualitative information, relevant data from qualitative sub-studies [13,15,16,17,18] were synthesized.

### 2.6. Analysis

We used descriptive analysis stratified by country and DAT type using R 4.3.3 and Stata 18. For platform logins, the percentage of weekdays in the main enrollment period where there was at least one visit to the adherence platform at each facility was calculated. For SMS reminder indicators, the number of treatment days was calculated for each participant. DAT engagement indicators were represented by digitally recorded doses by the participants and doses manually added by the HCP. Participants were analyzed primarily based on the DAT they initiated treatment with. Platform data were restricted to the trial’s main enrollment phase and the associated 6-month follow-up period whilst PwTB were on treatment or 12 months for the Ethiopia trial. We conducted a content analysis of the qualitative sub-studies guided by the process evaluation indicators. Indicators were grouped by thematic similarity to identify related content in each qualitative sub-study.

## 3. Results

### 3.1. Summary of Inputs

The DAT intervention was implemented in 162 facilities across the five countries, enrolling 10,377 participants starting a DAT in the intervention arms. The average number of healthcare providers trained per facility ranged from two to seven across the five countries (Table 2). Training before the intervention initiation was a condition for proper DAT implementation in healthcare facilities. HCPs noted that staff shortages caused by COVID-19 impeded the effective multiplication of training by peers who received implementation training. In the Philippines, HCPs mentioned wanting more training sessions and staff shortages making it difficult to properly instruct colleagues.

“I wish there would be another training so that I can really know the program. I was just able to do that by fiddling with my cellphone.”

—HCP in the Philippines

Cellphone ownership (not shared) among PwTB was highest in South Africa (90% pillbox, 97% medication labels) and lowest in the Philippines (63% pillbox, 64% medication labels). In Ethiopia and Tanzania, it was similar across DAT types, ranging from 68% to 72%. For the implementation of medication labels, having access to a cellphone was a condition to be offered a DAT. The expansion of mobile coverage and cellphone usage in Ethiopia was reported as a facilitating factor for DAT usage. It was not uncommon for people to share a cellphone with other family members. People would stop using the medication labels or change to a pillbox if their cellphone was damaged or lost.

“That’s when we have a problem when the patient doesn’t have a cell phone and they don’t have a support system, so we give them a pillbox.”

—HCP in the Philippines

### 3.2. Intervention Fidelity

Process indicators are summarized in Table 3. The total SMS reminders sent per PwTB was highest in South Africa and Ethiopia (60.7 and 53.1/PwTB) and lowest in Tanzania (30.9/PwTB). The percentage of days on treatment during which participants received same-day reminders was higher among participants using labels versus pillboxes in Ethiopia (32% vs 23%), the Philippines (38% versus 18%) and South Africa (37% versus 21%), and similar for Tanzania (15% versus 13%). Reminders sent for a previous day’s missed dose showed similar differences by DAT type for the Philippines and South Africa.

Individual reasons prompting reminder messages from the platform included forgetting to send a message when a dose was taken or forgetting to take the dose before the predefined time. Other factors prompting reminder messages were inability to read, technology fatigue, lack of understanding of DAT usage/purpose, and low technology savviness. The latter was noted among the elderly population who were interviewed.

Intermittent access to cell phones and needing to have a positive airtime balance to send messages also influenced the ability to send messages as instructed. Technical issues such as pillboxes running out of battery and unstable network and electricity supply also played a role in medication intake information not being registered in timely fashion on the platform. As a result, in some instances, the adherence platform may have sent reminder messages even when medication was taken and DATs utilized correctly.

“Sometimes I receive a message that says, “you did not take your medicine today.” At that time, I came here [to the health facility] and explained that I have taken the medicine, but my house has a network problem. This inconvenience happens because of network, not because I didn’t take it.”

—PwTB in Ethiopia

Among participants who initially received the labels, the percentage who switched to the pillbox varied from 1% (8/1141) in Ethiopia to 35% (26/74) in South Africa. Among individuals enrolled from facilities randomized to the label arm, 11% of participants started treatment using a pillbox in Ethiopia, while in the Philippines, Tanzania, and South Africa, this percentage was 19%, 46%, and 91%, respectively. In South Africa, the label intervention was stopped early due to multiple problems with implementing the SMS component and the facilities transitioned to the pillbox intervention (see Supplementary Materials Section S4.1).

Participants switching from medication labels to smart pillboxes were mentioned in the Philippines, Tanzania, and South Africa qualitative studies. Inability to read, technology fatigue, lack of familiarity with cellphones, and forgetting to send an SMS were some of the reasons that influenced individuals to switch DATs. Issues such as technical glitches, leading to excessive SMS reminders received, or not being able to send the SMS code also influenced the decision to switch from the medication labels to the smart pillbox. Cellphone damage or loss, the requirement of having positive airtime balance, recurrent power cuts, and poor network connections were also mentioned as drivers of switching DATs.

“I didn’t use the stickers [medication labels] during that time because I had lost my phone, do you get me? I arrived there and told the sister [TB nurse], that’s when they gave me the box.”

—PwTB in South Africa

Tanzania had the highest number of home visits per participant using a DAT recorded on the adherence platform, at 25% (576/2339). This differed by DAT type: 8% (127/1656) for pillbox and 66% (449/683) for medication label. The practice of home visits in Tanzania was supported by a large cadre of community health workers, and they were part of routine care prior to the implementation of the intervention. In South Africa, 8% (153/1834) of participants had a home visit, 69% (51/74) of those using the labels, and 6% (102/1754) using the pillbox. The percentage of participants who had home visits in Ethiopia, the Philippines, and Ukraine was ≤1%.

Qualitative data indicated that in all countries other than Tanzania, staff shortages limited the number of home visits conducted. In Ukraine, home visits were not frequent due to the healthcare reform that led to staff reductions in specialized TB facilities and displacement of both PwTB and HCPs due to the war. Stakeholders’ interviews indicated that stigma-related issues influenced the feasibility of home visits. It was reported that some individuals would deliberately provide wrong addresses to avoid receiving visits.

“Patients do not want to be visited to their home as there are TB patients whose families do not know that they have TB.”

—HCP in Ethiopia

Homelessness, not having a fixed address, relocations, or living in informal settings without an official address were also barriers to HCPs conducting home visits.

**Figure 1:**
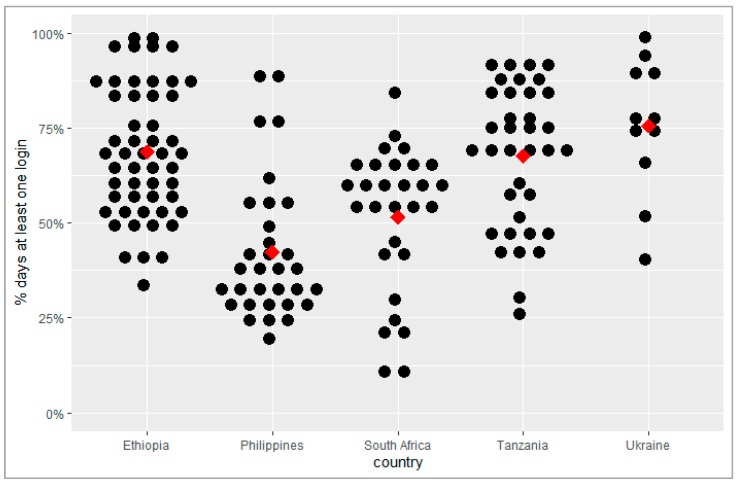
Percentage of weekdays with at least one login to the adherence platform per facility. Each black dot represents a facility; the arithmetic mean is indicated by the red diamond.

The percentage of weekdays with at least one login to the adherence platform per facility ranged from 42% in the Philippines to 76% in Ukraine (Table 3). It was 52% in South Africa, 68% in Tanzania, and 69% in Ethiopia (Figure 1). The mean number of minutes per facility per day spent on the platform was highest in Tanzania, at 18 min, and lowest in the Philippines, at 4 min.

HCPs from the Philippines, South Africa, and Tanzania reported that the adherence platform reduced their workload and simplified activities. They were able to monitor multiple users’ treatment adherence at once.

“This new system (adherence platform) has simplified our work, for instance when you enter the office in the morning, you look on the tablet to monitor patients’ treatment adherence, make follow up on patients’ with bad adherence.”

—HCP in Tanzania

However, some HCPs also perceived that the platform added to their workload. In the Philippines, the adherence platform, and the local Integrated Tuberculosis Information System (ITIS) operated simultaneously, requiring HCPs to input similar users’ information into both systems, increasing workload. In Ukraine, HCPs considered the process of validating dosing records burdensome.

“But look, if the program (adherence platform) already recorded that the dose is missed, why does it have to be colored (verified) again? It is logical, right?”

—HCP in Ukraine

Three types of devices were used to access the adherence platform: a tablet (provided by the ASCENT project), desktop or laptop computer, or a smartphone. In Ethiopia and Tanzania, 89% of logins to the platform were from a tablet, while this figure was 93% in South Africa. In the Philippines and Ukraine, most logins were from smartphones.

### 3.3. Intervention Coverage

As shown in Table 4, the percentage of PwTB starting a DAT ranged from 55.2% in Ukraine to 73.5% in South Africa. The pillbox arm had both the lowest DAT coverage at 51.4% in the Philippines and the highest at 79.8% in South Africa. Digitally recorded doses were higher in the smart pillbox arm versus the label arm. The percentage of total doses digitally recorded ranged from 82% in Ukraine to 91% in Tanzania among pillbox users and 62% in South Africa and 84% in Tanzania among label users. The Philippines had the highest percentage of manual doses added to the adherence platform more than 7 days after the dose day and when a dose was missed in both pillbox and label arms at 61% and 55%, respectively.

Over 96% of sub-study 1 survey respondents in Ethiopia and Tanzania agreed that using a pillbox DAT made them feel more connected to their HCP. In the Philippines, 70% of labels users reported better connection to their HCP, while this was over 95% in Ethiopia and Tanzania, with 86% of label users in South Africa agreeing.

According to HCPs, the adherence platform assisted them to act when they observed a PwTB not engaging with the DAT, thereby strengthening communication between the two parties. Some trust issues also emerged mediated by DAT technology. HCPs expressed skepticism on the proper usage of the DATs: they believed that certain PwTB were using the DATs without taking their medication to avoid being contacted by HCPs, which negatively impacted their relationship.

“Well, it’s clear that if you are a person having a smart pillbox and the doctor calls you 10 times in a month, you’ve already opened the smart pillbox so that the doctor doesn’t bother you, doesn’t call you. And we have some (meaning patients) who say to us: you are so concerned about my health... Because we call them often.”

—HCP in Ukraine

## 4. Discussion

In this process evaluation, we found that engagement with DATs was high among participants, particularly in the pillbox arm, with digitally recorded doses ranging from 82% to 91% and strong concurrence from the qualitative interviews. There was considerable variation in both method and frequency of HCPs accessing the digital adherence platform, but their overall engagement was high, as confirmed by manual dosing whenever digital recording was missed. Phone ownership was high overall, but it varied by country.

The findings confirm the pragmatic nature of the trials, with each country adapting interventions to their local context. Moreover, this study provides a rich diversity of context for the implementation of DATs in terms of geographical, cultural, socioeconomic, and demographic factors, and has practical implications for implementation. Key findings indicate that integration of digital systems into daily workflow is complex, and the ability of health systems to adopt the new technology varies per country and facility. Health system and HCP capacity has an impact on the ability to utilize real-time data. Reviewing adherence data daily may not be possible for all HCPs or necessary for all PwTB, and value may still be derived from less regular assessment of adherence and acting where doses are missed, or prioritizing adherence monitoring of a certain population. DATs are a highly acceptable form of treatment support for PwTB and for the HCPs who can integrate digital systems with their regular tasks.

Overall, DAT engagement was high, measured by the percentage of digitally recorded doses. Label users recorded between 62% and 84% of doses by texting the toll-free number. Conversely a study in Uganda where self-report of dose taking was accomplished by calling 99DOTS was 58%, dropping further over the course of treatment [27], while a meta-analysis on DAT projects across ten countries showed that overall average adherence among people with DS-TB varied between 80% and 90% [28]. Engagement of pillbox users was higher than labels, with 82–91% of doses digitally recorded (Table 4). Sustained engagement offers a measure of reach of the intervention, and high levels indicated users like DATs. A positive association between treatment satisfaction and medication adherence and higher levels of treatment satisfaction among participants using a pillbox versus standard of care was previously reported [29]. Similarly, the ASCENT trials, a study on feasibility and acceptability, showed that nearly all DAT users reported that using DATs motivated them to complete treatment [13]. Furthermore, a recent scoping review suggested that video and pillbox DATs are generally acceptable with moderate to high levels of engagement [30].

Access to a mobile phone was a requirement for using the medication label and the intervention can be implemented with a shared phone, although unintended disclosure of TB status is a potential risk (Table 2). Access to mobile technology is key to the implementation of digital health interventions [31]. It was found that those with the highest engagement with 99DOTS were those who owned a mobile phone that was not shared [32]. Timely delivery of differentiated care is facilitated by a phone that is not shared. Providing affordable mobile phones to PwTB could be considered part of the strategy to enhance the use of DATs for treatment support.

Variation in fidelity of the intervention may be due to differences in the cadre of staff trained and intensity of training received. In Ukraine, for example, TB doctors would enroll PwTB on DATs, while in the Philippines, due to redeployment to COVID-19 services, additional staff from other disciplines received on-the-job training from TB focal persons, enabling them to enroll and monitor PwTB. The lack of more comprehensive training of the staff providing secondary support in the Philippines may have impacted their ability to implement the intervention as planned. In addition, it is likely there were varying skill levels in the use of digital technology and in building supportive relationships with DAT users. Access to regular retraining and ensuring a strong knowledge base on DATs at facility level may mitigate the effects of staff turnover and varying digital literacy levels.

Variation in HCP engagement with the adherence platform may be influenced by several factors. A higher percentage of weekdays with at least one login to the platform per facility indicates that use of the platform was integrated into routine workflow. It is possible that with more staff trained, as in Ukraine, there was collective rather than individual action to make checking the platform routine practice, seen as key to professional behavior change in complex healthcare settings [33]. Daily reviewing of DAT data was indicated by 90% of HCPs in a meta-analysis of six studies [32], although this was a small sample self-reporting rather than verified through automated platform statistics as in this study. A cluster-randomized trial in China concluded that HCPs did not use the electronic adherence data as intended, possibly due to frequent reports of pillboxes failing to log doses resulting from battery issues, although with a different platform and DAT [34]. The substantial number of days with no facility staff accessing the adherence platform observed in South Africa and the Philippines could be explained by high workloads or lack of motivation (Table 3). A lack of trust in the adherence data due to difficulty identifying those truly experiencing adherence issues may undermine staff motivation to use DATs [35,36]. Although qualitative data indicated the use of DATs reduced workload, a finding also emphasized in other studies [37], it is possible that for HCPs who are already overburdened and lacking digital skills, daily review of adherence may not have been feasible.

The existence of an electronic patient system, as in the Philippines and Ukraine, in addition to the adherence platform may be a facilitator or a barrier to the intervention. Users of e-TB Manager in Ukraine gave high ratings to the system’s ability to improve case management [38]. TB staff were already accustomed to using digital systems and had put in the time required to embed the behavior change. However, they may also see it as additional work if they need to log in to two separate systems for PwTB management, a finding from qualitative interviews with HCPs in Ukraine. Direct integration of adherence data into existing digital systems could mitigate this and make utilization of real-time adherence data more seamless.

DAT coverage differed among countries (Table 4), and the factors contributing to uptake of DATs were varied. Staffing issues such as rotation in Tanzania or redeployment in Philippines, frequent power outages in South Africa, and healthcare reform as well as the war in Ukraine impacted coverage. Comparable results were reported in a Ugandan trial with 52% coverage [5]. Lower numbers of PwTB starting treatment with a DAT and variability over time underline the pragmatic nature of the trial, where routine staff implement the intervention.

Manual dosing was an important part of the intervention and provided a measure of HCP engagement (Table 4). The addition of manual doses to the adherence platform more than seven days after a missed dose was less than 30% for both DAT types in Ethiopia and greater than 50% for both Philippines and Ukraine. HCPs would need to contact the PwTB to confirm if the dose was taken or not before adding a manual dose to the platform. Adding a manual dose within a short time of a missed dose may indicate a systematic approach to monitoring adherence, even if the capacity of the facility was low. Failure to add a manual dose within 1 week of a missed dose may indicate poor implementation or lack of engagement by HCPs with the intervention, in addition to low capacity at the facility. It could also be due to the inability to contact the PwTB. In Ukraine, the addition of manual dosing decreased significantly from pre-war to during the war, indicated by an increase of 7.8% to 30% of overall treatment days showing no information [39].

The feeling of connection to their HCP was high among participants in most countries (Table 4), a finding that concurs with that of previous studies [40]. It has been shown that better adherence to treatment may be facilitated by good PwTB–provider relationships [27,41,42] and the feeling of being cared for may be enhanced by DATs. However, this was lower in Philippines, particularly for label users [15]. This finding concurs with other results from the Philippines, indicating that HCPs had difficulty implementing the intervention as planned.

This research provides evidence about the process by which DATs were implemented in real-world settings across five diverse contexts, advancing the current knowledge. Variation in fidelity of DAT implementation was evident, and likely due to several factors at individual, local, and national levels. Firstly, the level of experience and expertise of staff trained on DATs varied. Secondly, at facility level, there were differences in frequency of HCPs accessing adherence and using adherence data to monitor PwTB, and thirdly, a greater proportion of PwTB owned their own mobile phones in South Africa compared to the Philippines, while DAT coverage was impacted by staffing rotation and redeployment in all countries. Ensuring implementation would require establishing benchmarks for fidelity, monitoring, and using a quality-assurance feedback system at the national level. At a local level, adaptation of the DAT intervention may be required to allow staff to work according to their own judgment of what fits the PwTB and local context, such as offering a choice of DAT to those who could most benefit most or utilizing alternative differentiation-of-care approaches. Furthermore, comprehensive regular training of a wide cohort of staff to deliver differentiated care and integrating adherence data with electronic patient management systems would facilitate fidelity to the intervention.

A limitation of the study is that qualitative results are from secondary data collected for various sub-studies. The sub-studies were designed to evaluate feasibility and acceptability of DATs through surveys and HCP and PwTB in-depth interviews, with a sub-sample of the trial participants, with a small sample per country. The quantitative data we collected were from logs, reports, and platform usage statistics. We did not conduct observations of work practices, which could have further enhanced understanding of implementation. Analysis was conducted by country and DAT type. Facility-level analysis could provide useful insights into differences in implementation within countries. Furthermore, the impact of contextual factors was not considered, such as technology or infrastructure downtime or participants opting out of daily SMS reminders. This omission was due to a lack of available data.

To our knowledge, this is the first process evaluation of a digital adherence technology intervention in tuberculosis implemented in diverse contexts. A strength of this study is the comprehensive assessment of the DAT interventions across several components. Measurement of many of the process indicators was based on data captured objectively by the adherence platform. A further strength of this research is its mixed-method approach, data being obtained from multiple sources, including related sub-studies, which were integrated to form an overall result. The synthesis of both quantitative and qualitative evidence provides a comprehensive account of how DATs were implemented.

## 5. Conclusions

In conclusion, we observed variation in the level of fidelity to and coverage of the intervention across the five countries. Daily utilization of real-time data to monitor adherence and subsequent performance of actions required for differentiated care was impacted by the capacity of the health systems and staff working within them. Engagement with DATs was high and they were found to be acceptable, facilitating enhanced connection with HCPs for engaged PwTB. DATs offer a valuable person-centered tool for supporting PwTB through treatment. Coverage and fidelity of digital support interventions are influenced by factors at the health system, facility, and individual levels. Future research could usefully focus on identifying optimal strategies to integrate digital systems to include adherence data and engage HCPs on its timely usage.

## Figures and Tables

**Table 1:** Summary of process evaluation indicators and their data sources.

	Indicator	Data Sources	Content Analysis Categorization
	Input		
1	No. of healthcare providers that received implementation training during the pilot and main enrollment period	Country training report	HCP implementation training
2	No. of implementation support visits made to health facilities by ASCENT staff during the main enrollment period	Facility visits and call logs	NA
3	Percentage of PwTB who own a mobile phone which is not shared	ASCENT sub-study 1 ^a^	PwTB with access to or own a mobile phone
	Process		
4	No. of automated SMS reminders sent by adherence platform per PwTB enrolled during the main enrollment period	Adherence platform SMS log	Automated SMS reminders sent by adherence platform
5	Percentage of treatment days with an automated SMS reminder sent by the adherence platform	Adherence platform SMS log	
6	No./percentage of PwTB that switched from medication label to smart pillbox during their treatment	Adherence platform PwTB record	PwTB switch from medication labels to smart pillbox during treatment
7	No. of home visits per PwTB by initial DAT received during the main enrollment period.	Adherence platform HCW action report	Home visits during the main enrollment period
8	No./percentage of PwTB who were shown their adherence data at the facility by the HCP	ASCENT sub-study 1 ^a^	NA
9	Total number of adherence platform logins per facility during main enrollment period	Adherence platform usage record	NA
10	Percentage of weekdays in the main enrollment period where there was at least one visit to the adherence platform at facility level	Adherence platform usage record	HCP interaction with the adherence platform
11	Average minutes a HCP was logged onto platform per day	Adherence platform usage record	
12	Average percentage of ASCENT tablets used to access adherence platform during main enrollment period	Adherence platform usage record	
	Output		
13	Percentage of PwTB who started a DAT in the main enrollment period	Adherence platform PwTB record and facility TB register	NA
14	Average percentage of doses digitally recorded of all doses recorded during main enrollment period	Adherence platform dosing report	NA
15	Patterns of manual doses added to the adherence platform more than 7 days after the original date during main enrollment period	Adherence platform dosing report	NA
	Outcome		
16	Can the use of DATs and differentiated care influence PwTB–HCP relationship?	ASCENT sub-study 1 ^a^, 2 ^b^ and 3 ^c^	People with TB–HCP relationship

^a^ Sub-study 1: cross-sectional survey to assess the acceptability and feasibility of the interventions for people with TB (conducted in all countries except Ukraine); ^b^ sub-study 2: in-depth interviews of people with TB to assess the acceptability and feasibility of DATs and differentiated care (conducted in all countries except Ukraine); ^c^ sub-study 3: in-depth interviews of healthcare workers and key stakeholders’ perspectives to assess the acceptability and feasibility of DATs and differentiated care (conducted in all countries). NA: not applicable; PwTB: people with TB; SMS: short message service; DAT: digital adherence technology; HCP: healthcare provider.

**Table 2:** Results of indicators assessing implementation inputs by country and DAT type (where relevant).

	Indicator	Ethiopia	Philippines	South Africa	Tanzania	Ukraine
1	Number of HCPs trained/facility ^a^	3.0 (155/52)	4.9 (157/32)	2.6 (79/30)	2.0 (72/36)	7.4 (89/12)
2	Number of implementation support visits/facility ^b^	1.7 (89/52)	2.4 (78/32)	1.3 (39/30)	1.8 (65/36)	0.7 (8/12)
3	Phone ownership (not shared) among PwTB using the pillbox ^c^	72% (36/50)	62% (31/50)	90% (44/49)	70% (43/61)	NA
	Phone ownership (not shared) among PwTB using the labels ^c^	70% (35/50)	64% (33/52)	97% (28/29)	68% (26/38)	NA

HCP: healthcare provider; PwTB: people with TB; NA: not applicable. ^a^ training log; ^b^ implementation support visit log; ^c^ data from sub-study 1.

**Table 3:** Results of indicators assessing implementation processes by country and DAT type (where relevant).

	Indicator	Ethiopia	Philippines	South Africa	Tanzania	Ukraine
	Number of participants starting a DAT in the main enrollment phase overall and by initial DAT received: total (pillbox/labels/unknown)	2518 (1375/1141/2)	2844 (1472/1370/2)	1834 (1754/74/6)	2339 (1656/683/0)	842 (842/NA/0)
	**Automated SMS reminders:**					
4	Number of SMS reminders sent ^a^ (number of reminders per participant)	133,615 (53.1)	130,802 (46.0)	111,237 (60.7)	72,188 (30.9)	NA
5	% treatment days same-day reminder was sent—pillbox	23% (46,560/205,291)	18% (33,939/193,732)	21% (66,359/310,157)	13% (31,889/243,261)	NA
	% treatment days same-day reminder was sent—labels	32% (54,263/169,238)	38% (52,565/139,513)	37% (4792/12,975)	15% (14,136/96,605)	NA
	% treatment days previous-day reminder was sent—pillbox	8% (16,319/205,291)	9% (18,117/193,732)	12% (37,394/310,157)	8% (18,518/243,261)	NA
	% treatment days yesterday reminder was sent—labels	10% (16,473/169,238)	19% (26,181/139,513)	21% (2692/12,975)	8% (7645/96,605)	NA
	**DAT type summary**					
6	% switch from labels (initial DAT received) to pillbox	1% (8/1141)	5% (63/1370)	35% (26/74)	14% (96/683)	NA
	% started on pillbox in labels arm ^b^	11% (147/1287)	19% (329/1264)	91% (736/812)	46% (572/1253)	NA
	**Home visits**					
7	# home visits, as % of # enrolled (platform)	0.4% (11/2518)	0.6% (17/2844)	8% (153/1834)	25% (576/2339)	0.4% (3/842)
	# home visits, as % of # enrolled (platform)—pillbox	1% (11/1375)	0% (0/1472)	6% (102/1754)	8% (127/1656)	0.4% (3/842)
	# home visits, as % of # enrolled (platform)—labels	0% (0/1141)	1% (17/1370)	69% (51/74)	66% (449/683)	NA
	**PwTB shown adherence data**					
8	% of participants shown platform data—pillbox ^c^	64% (32/50)	46% (23/50)	94% (46/49)	80% (49/61)	NA
	% of participants shown platform data—labels ^c^	70% (35/50)	44% (23/52)	93% (27/29)	76% (29/38)	NA
	**Platform Usage (by HCP)**					
9	# platform visits (this may include multiple logins per day from the same user)	37,310	10,542	20,573	51,768	9608
10	% of weekdays with at least one login to adherence platform ^d^	69%	42%	52%	68%	76%
11	Arithmetic mean minutes spent on the platform per day	5.5	4	11	18	13
12	% tablet used to access the platform	89%	38% (53% smartphone)	89%	93%	8% ^e^ (36% desktop; 56% smart phone)

All data from the adherence platform unless indicated otherwise. NA: not applicable; PwTB: people with TB; SMS: short message service; DAT: digital adherence technology; HCP: healthcare provider; # number. ^a^ The total includes both reminder messages to take treatment if a dose was not confirmed on the platform after a set time and a reminder sent if the previous day’s dose was missed, expressed in parentheses as the number of reminders/PwTB started on a DAT. ^b^ Denominator is the number of PwTB who started a DAT in facilities allocated to the label arm. ^c^ Data from sub-study 1. ^d^ Defined at the facility level as the number of days with at least one login to the adherence platform/number of weekdays in the main study phase × 100. At the country level, this is summarized as the arithmetic mean of facility-level percentages. ^e^ Ukraine did not distribute tablets to HCPs. Numbers in parentheses are the numerator and denominator of the percentage presented.

**Table 4:** Results of indicators assessing implementation outputs and outcomes by country and DAT type (where relevant).

	Indicator	Ethiopia	Philippines	South Africa	Tanzania	Ukraine
	**DAT Coverage**					
13	% of PwTB who started a DAT in the main enrollment period	NA ^a^	61.8% (2844/4604)	73.5% (1834/2494)	66.0% (2339/3546)	55.2% (842/1526)
	% of PwTB who started a DAT in the main enrollment period—pillbox	NA ^a^	51.4% (1150/2236)	79.8% (1022/1281)	61.1% (1086/1778)	55.2% (842/1526)
	% of PwTB who started a DAT in the main enrollment period—labels	NA ^a^	71.5% (1694/2368)	66.9% (812/1213)	70.9% (1253/1768)	NA
	**DAT Engagement**					
	Number of PwTB—pillbox	1375	1472	1754	1656	842
14	% digital recorded doses—pillbox	90% (177,599/196,352)	83% (172,234/208,130)	88% (207,569/235,417)	91% (202,282/222,076)	82% (91,712/111,901)
15	% of manual doses added >7 days after the dose day—pillbox	30% (5075/16,845)	61% (14,708/24,015)	44% (3966/9075)	32% (4623/14,561)	52% (6858/13,206)
	Number of PwTB—labels	1141	1370	74	683	-
14	% digital recorded doses—labels	81% (126,718/156,832)	69% (119,304/171,786)	62% (3815/6154)	84% (63,862/76,231)	NA
15	% of manual doses added >7 days after the dose day—labels	21% (6016/28,782)	55% (20,832/37,919)	47% (541/1149)	26% (2906/11,108)	NA
	**Outcome**					
16	% participants agreeing that using DAT made them feel more connected to their HCPs—pillbox ^b^	100% (50/50)	84.0% (42/50)	91.8% (41/49)	96.7% (59/61)	NA
	% participants agreeing that using DAT made them feel more connected to their HCPs—labels ^b^	98.0% (49/50)	69.2% (36/52)	86.2% (25/29)	94.7% (36/38)	NA

All data from the adherence platform unless indicated otherwise. ^a^ Not applicable for the Ethiopian CRT, as the study design differed. Data were only collected on people with TB who consented to the study; ^b^ data from sub-study 1. DAT type missing for 10 individuals (2 Philippines; 6 South Africa; and 2 Ethiopia). NA: not applicable; PwTB: people with TB; DAT: digital adherence technology; HCP: healthcare provider.

## Data Availability

The raw quantitative data supporting the conclusions of this article will be made available by the authors without undue reservation. Selected quotes from qualitative interviews are in the manuscript.

## Supplementary Materials

The following supporting information can be downloaded at https://www.mdpi.com/article/10.3390/tropicalmed10030068/s1. Figure S1: Process evaluation framework; Table S1: Data sources; Table S2: Participant demographics; Figure S2: Logins to adherence platform by country and facility; Figure S3: DAT coverage by country.

## Abbreviations

The following abbreviations are used in this manuscript:

ASCENT	Adherence Support Coalition to End TB
CRT	cluster-randomized trial
DAT	digital adherence technology
DS-TB	drug-sensitive tuberculosis
HCP	healthcare provider
PwTB	person with tuberculosis
RCT	randomized controlled trial
SMS	short message service
TB	tuberculosis
RCT	randomized controlled trial
WHO	World Health Organization
