# Supplementary material for: Process Evaluation of Pragmatic Cluster-Randomized Trials of Digital Adherence Technologies for Tuberculosis Treatment Support: A Mixed-Method Study in Five Countries
Source: Trop Med Infect Dis. 2025 Mar 6;10(3):68. doi: 10.3390/tropicalmed10030068 (PMC11946463; doi:10.3390/tropicalmed10030068)
Supplement: Supplementary file 1 [file tropicalmed-10-00068-s001.zip › tropicalmed-3458102-supplementary.pdf]

Supplementary Information

For Madden et al “Process evaluation of pragmatic cluster randomized trials of digital adherence technologies for tuberculosis treatment support: a mixed methods study in five countries”

1 Methods 2
1.1 Trial and sub-studies 2
1.1.1 Trials 2
1.1.2 Sub-study 1 2
1.1.3 Sub-studies 2 and 3 2
1.2 Process evaluation framework and overview of indicators 3
1.3 Data Sources 4
2 Context for Delivery 6
2.1 Infrastructure 6
2.2 Task Lists 7
2.2.1 Platform – what HCP can see/use: 7
2.3 SMS 7
2.4 Training of HCPS 8
2.5 Implementation support 8
3 Results 9
3.1 Additional results: 9
4 Additional information for South Africa 11
4.1 Implementation challenges of the sleeve-label intervention in South Africa 11

1 Methods

1.1 Trial and sub-studies

1.1.1 Trials

Countries were chosen to reflect varied epidemiology and healthcare settings. Clusters were health facilities or rayons in Ukraine. In the Philippines, South Africa, Tanzania, and Ukraine, clusters were randomised to the DAT intervention or standard of care (ratio 1:1) arms, and a second randomisation allocated intervention facilities in the Philippines, South Africa, and Tanzania to either a pillbox DAT or medication labels DAT. In Ukraine, only pillboxes were used due to the loose drug format. In Ethiopia, clusters were randomised to pillbox DAT or medication labels DAT or standard of care (ratio 1:1:1) arms. PwTB in clusters allocated to medication labels with no access to a mobile phone were offered the pillbox instead. TB staff at the health facilities/rayons implemented the intervention, with support from interns in South Africa.

1.1.2 Sub-study 1

In each country, except Ukraine due to the war, a facility-based cross-sectional survey of 10 health facilities implementing the DAT interventions with differentiated care was conducted to assess the acceptability and feasibility of the interventions for people with TB. Facility selection ensured the inclusion of urban/rural and public/private facilities. Quantitative data were collected from 10 randomly selected PwTBs per facility who started to use the DAT. PwTB selection ensured the inclusion of both male/female and intensive/continuation phases of TB treatment.

1.1.3 Sub-studies 2 and 3

In-depth interviews were carried out as part of two sub-studies assessing the acceptability and feasibility of DATs and differentiated care. The first study concentrated on assessing PwTB's experience, while the second one centred on healthcare workers and key stakeholders perspectives in the five countries. Due to the wartime conditions in Ukraine, PwTB interviews were not conducted. For each country and DAT group, around 20 individuals were interviewed. The interviews encompassed various aspects of PwTBs' experience with DATs and differentiated care, including factors that affect technology usage, treatment adherence, as well as the impact of societal influences on technology utilization.

1.2 Process evaluation framework and overview of indicators

The process evaluation framework is summarised in Figure S1.

Quantitative process evaluation indicators were identified for “input”, “process”, “output” and “outcomes” components of the DAT intervention and supplemented with contextual factors from content analysis of qualitative data. See Table 1 main paper.

Input indicators focused on DAT training and support for healthcare providers (HCPs), and mobile phone access by people with TB (PwTB). Process indicators primarily concentrated on adherence platform data such as treatment days with an automated SMS reminder, percentage of doses manually recorded, and adherence platform logins per facility. Output indicators also focus on platform data for digital dosing and patterns of consecutive manual dosing added >7 days after the scheduled dose-day. The outcome indicator was based on quantitative and qualitative sub-study data from PwTB and healthcare providers about whether of intervention improved the PWTB-HCP relationship.

Intervention impacts (improvements in treatment outcomes including reductions in losses to follow-up, treatment failure, and recurrence in the Ethiopian trial) are assessed through the cluster randomised controlled trials, and thus not included in this framework, which documents only the lower stages of the pathway.

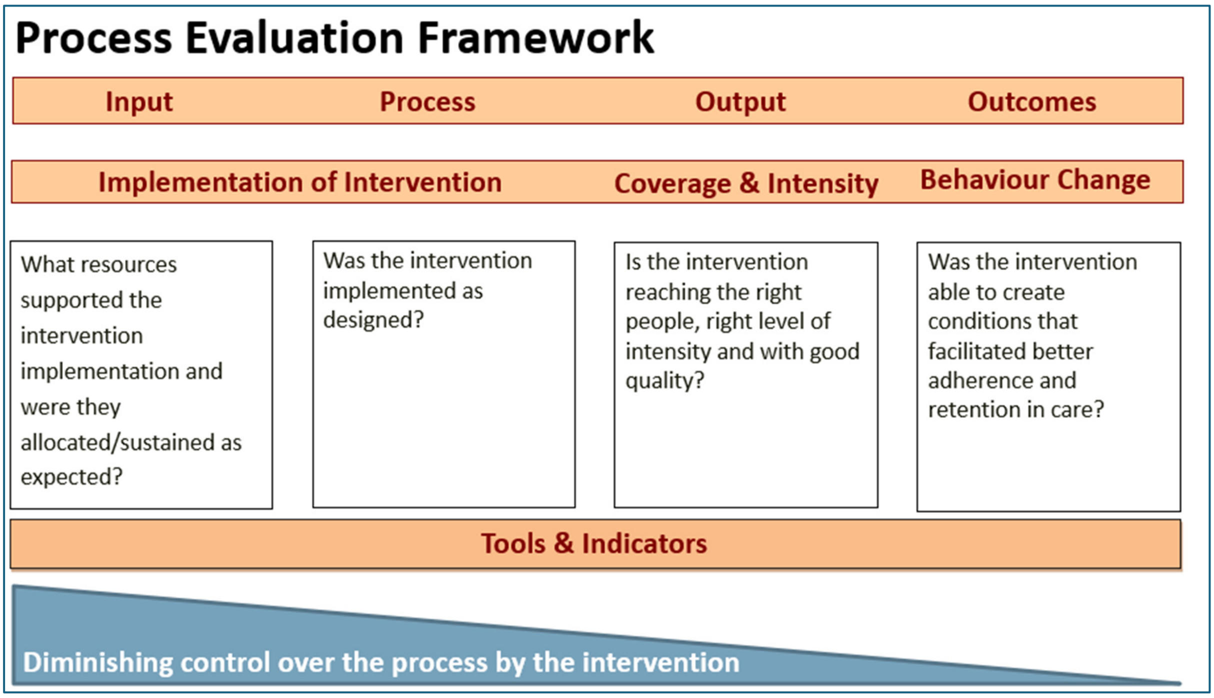

Figure S1: Process Evaluation Framework

Intervention impacts (improvements in treatment outcomes including reductions in losses to follow-up, treatment failure, and recurrence in the Ethiopian trial) are assessed through the cluster randomised controlled trials, and thus not included in this framework, which documents only the lower stages of the pathway.

1.3 Data Sources

Multiple data sources were used. Support actions performed by the health care provider (HCP) based on the differentiated care pathway, were logged on the adherence platform. Support actions included phone calls to the PWTB, facility visits by the PWTB or home visits by the HCP. Free text notes and tags could also be added for each PWTB. An SMS log recorded automated messages sent to DAT PwTBs. Healthcare provider engagement with the adherence platform was measured from automatically generated platform statistics. Date and time, duration, access device, and number of actions performed per unique platform user was available.

Table S1: Data Sources

Data Source	Dataset	Description
ASCENT adherence platform	PwTB Report	Collects PwTB contact details to operationalise the intervention
	Adherence/DAT engagement report	Record of each dose day per PwTB on a DAT for adherence monitoring
	Actions	Log of support actions taken by HCP including phone call, facility visit by PwTB or home visit by HCP
	SMS	Log of message sent and received per DAT
	Platform usage statistics	Platform usage statistics - Date and time, duration, access device, and number of actions performed per PwTB on a DAT
Logbook	Facility visit log	Record of facility visits and support calls from ASCENT staff to facilities
Logbook	Training log	Number of sessions and participants attending DAT training
Sub study 1	Qualitative	Qualitative study on patient acceptability and costs
Sub Study 2	Acceptability and feasibility	Participant and providers acceptability and feasibility of DATs

2 Context for Delivery

2.1 Infrastructure

Operationalization of intervention

Operationalization of the adherence intervention consisted of the use of either a smart pill box or a medication sleeve or label, and an online adherence platform (the Everwell Hub), which both DATs were linked to. With the use of a plastic medication container, a battery powered, re-usable module and a mobile data connection, the smart pill box automatically logs medication intake each time the person on TB treatment opens the box to take medication by sending a signal to the adherence platform. The medication sleeve/label, also called 99DOTS, uses customized packaging such as printed sleeves or labels that fit Fixed Dose Combination (FDC) medication for drug susceptible TB treatment. The person on TB treatment reports medication intake daily, either by calling a toll-free phone number or sending a free SMS message using a code found on the package. DATs were procured by ASCENT and shipped to participating countries, they were distributed to each intervention facility. Both DATs are linked to an online adherence platform (the Everwell Hub). In all countries except Tanzania, the adherence platform was deployed using the Azure Global Cloud with data centers in South Africa and India. For Tanzania, where it was prescribed by MoH that platform and data need to be hosted in-country from the start, an adapted approach was developed.

Health care providers can log into a single portal, either via a web browser or dedicated application on smartphone or tablet. PwTB can be registered and followed up via the platform. The adherence platform shows which PwTB are currently on treatment using a DAT, their day of treatment and medication taking behavior on a calendar.

The infrastructure to deliver the intervention, training of HCPs, and support visits to health facilities made up the resources to support the implementation of the intervention. The infrastructure included the online adherence platform which both DATs were linked to, hosting of the platform in each country, hardware (tablets, desktops) and data services for HCPs to deliver the intervention. Training of HCPs implementing the intervention included formal training sessions and mentoring on the effective utilization of the adherence platform, PwTB monitoring, manual dosing procedures, and the provision of differentiated care. Training sessions were repeated, including for newly recruited staff. Additionally, HCPs from departments other than TB, who did not receive formal training, but were added to the cohort of staff who could initiate a participant on a DAT, received cascade and on-the-job training from the TB focal person who was formally trained previously. This style of training was utilized when core TB staff were redeployed to the COVID-19 response, or when capacity was low.

The study team trained HCPs on DATs, provided implementation support, and addressed technical issues with DATs on the adherence platform. In the preparatory phase that took place 3-6 months before the trial enrollment phase, the focus included facility readiness to implement the intervention and operationalizing the use of DATs. Stock levels of DATs were monitored during visits, as well as recording of previous stockouts. Defective or malfunctioning DATs were noted and replaced.

2.2 Task Lists

Information on adherence is automatically processed. The task list is a grouping of PwTB based on adherence. PwTB that fit certain criteria appear in a task list: 1) those who have missed yesterday's dose'; or 2) missed the last two doses or 3) missed three or more doses. Task lists were configured per country based on differentiation of care. Differentiated care actions (e.g., phone call or home visit) were proposed to be performed by the health provider as specific to the country context. Healthcare providers could record on the platform if they had taken follow-up action, they could also use the platform to record notes per PwTB. The Philippines was the only country where HCP received a daily SMS with the task list, this was a feature used previously in TB Reach and was maintained during ASCENT

2.2.1 Platform – what HCP can see/use:

- Task list – list of PwTB that need to be contacted based on differentiated care
- 2-week digital dosing summary, quick view – 10-15 PwTB/screen, 14-day calendar view
- Individual-level PwTB calendar view

2.3 SMS

If on a certain day, a PwTB has not taken their dose before a certain time, the adherence platform will automatically send a SMS reminder message to him/her on that day. It will also send a second SMS message to the PwTB the following day in case the dose was missed definitively.

Healthcare facilities in the intervention arm were provided with a tablet and data services to enable access. In Ukraine access was via desktop computers already in use at facilities. Healthcare provider engagement with the adherence platform is measured from automatically generated platform statistics. Datetime, duration, access device, and number of actions performed per unique platform user is available.

2.4 Training of HCPS

Training of health care providers was conducted in all countries. At least one healthcare provider was trained at each cluster, TB nurse or TB doctor. The training was face to face, took 2/3 days, and was conducted by local ASCENT staff. Training was divided into modules including platform, smart pillbox, and medication sleeve/label. Training on platform use provided step by step guidance on how patient registration; use of adherence calendar; adherence management; patient management; patient outcomes; and statistics and analysis.   Medication sleeve/label training included an introduction and overview of how the patient would use the medication sleeve; how to provide the medication sleeve/label to the patient; and how to resolve issues. Coaching and mentoring on DAT use and adherence platform use was provided to newly recruited staff outside of the formal training.

2.5 Implementation support

Project staff conducted regular site visits to support research and implementation activities. During the preparation phase, site visits focused on readiness for the intervention, operationalizing the use of DATs, and preparing SOPs and materials for research. DATs were delivered by digital technical officers, and research staff distributed research recording and reporting tools.

Challenges with implementation of the technologies was addressed, and technical issues resolved or escalated. Stock levels of DATs were monitored during visits, as well as recording of previous stock outs. Defective or malfunctioning DATs were noted. Tools for research recording and reporting were checked to ensure they were available and used as required, such as the Facility key Table forms, Social No harm registers, Informed consent forms. In addition to in person visits, site support was also provided through phone calls and messages on social media platforms throughout the period of implementation.

3 Results

3.1 Additional results:

Table S2: Participant Demographics

	Ethiopia	The Philippines	South Africa	Tanzania	Ukraine
Number of districts/regions/provinces	2 regions	2 provinces	4 regions	5 districts	2 provinces
Number of Intervention clusters: pillbox	26	16	15	18	12
Number of Intervention clusters: label	26	16	15	18	-
Eligibility criteria	Adults with pulmonary DS-TB	Adults with DS-TB	Adults with DS-TB	Adults with DS-TB	Adults with DS-TB
Total number of PwTB who started a DAT	2518	2844	1834	2339	842
Male	1334 (58%)	1879 (66%)	1139 (61%)	1522 (63%)	569 (67%)
Female	953 (42%)	965 (34%)	731 (39%)	899 (37%)	281 (33%)
Transgender	0 (0%)	2 (0%)	0 (0%)	0 (0%)	0 (0%)
Age (Years)	29 (23-40)	46 (32-58)	41 (33-51)	44 (33-59)	44 (36-54)

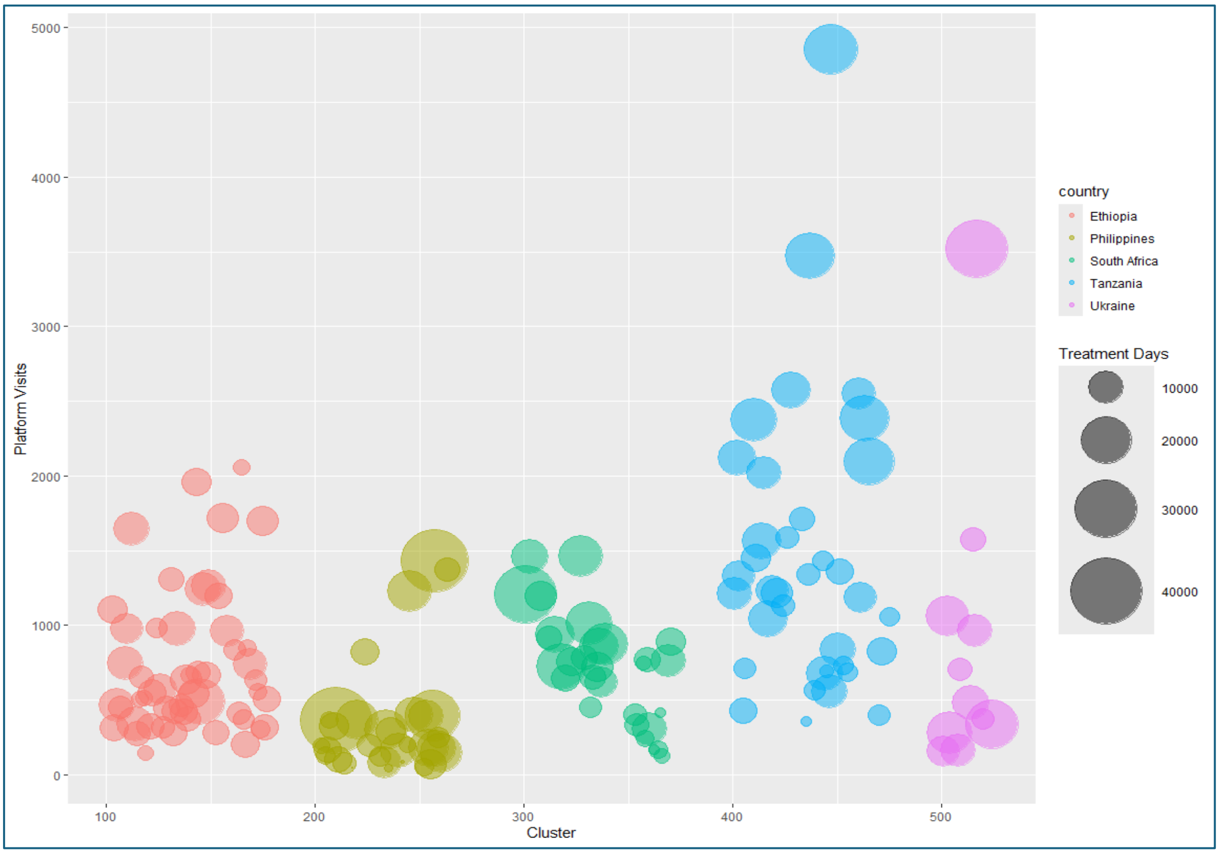

Figure S2: Logins to adherence platform by country and facility; the size of bubble represents number of treatment days.

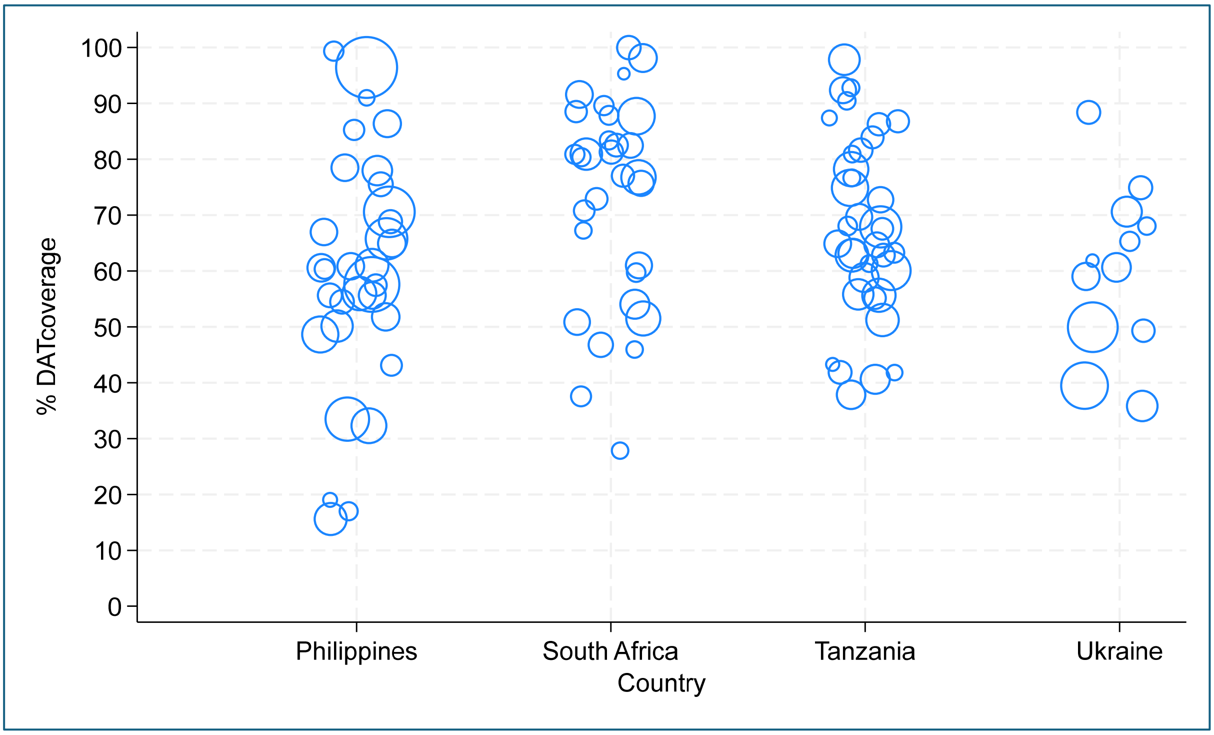

Figure S3: Each bubble represents a health facility; the size of the bubble is weight of the number of people starting TB treatment in that facility

4 Additional information for South Africa

4.1 Implementation challenges of the sleeve-label intervention in South Africa

In late 2021, after a careful review of data from the pilot phase in South Africa, a decision was made in South Africa to stop the labels intervention for new enrollees and implement the pillbox instead. The data were shared with our Technical Advisory Group (TAG) in August 2021 and the decision to stop the labels was supported by the TAG. A brief summary of the reasons are listed below.

1. **The sleeve/label arm could not be fully operationalized as intended**
   The requirements for the labels arm included that the use of short codes be: 1) accessible to all mobile phones with the mobile network carriers; 2) completely free to the user and 3) be used when the user has a zero or negative balance. These technological features were not achieved by the mobile providers in South Africa at the end of the pilot phase. As a result an important feature of the platform – sending SMS reminders after not digitally registering the dose – has been turned off for those using the sleeve/label DAT, which would have a huge impact on the intervention fidelity in the labels arm.
2. **Technological glitches in SMS reminders**
   We found that some participants had sent SMS short codes to register a dose taken that were not received by the Adherence platform. This resulted in inappropriately sending multiple reminder SMSs to participants. Anecdotal reports and interviews of health care workers and ASCENT study staff revealed high levels of frustration by the participants.
3. **Qualitative interviews in selected facilities in Tshwane District with interns and TB nurses documented challenges of delivering the labels intervention**
   To gain insights on the intermediate outcomes of implementation process of labels, we conducted qualitative interviews in selected facilities in Tshwane District with interns and TB nurses involved in the implementation of DAT.
   Challenges experienced by patients according to health workers were in two categories:

   Patient-level factors

   - Lack of understanding (participants see texting as needlessly burdensome)
   - Perceived high burden of texting "SMS fatigue"
   - Cell phone issues (patients not having a cell phone, phone sharing, no electricity to charge).

- Unable to text due to old age, illiteracy or being too sick

Technical issues

- Technical glitches (SMS does not go through to be recorded on the platform which leads to excessive reminder messages and frustration by patients), caused at the interface between cell phone and provider
- SMS not working due to requirement for Airtime by network provider

At the time of this analysis these problems were not observed to the same level as in South Africa, and therefore the labels intervention continued in Ethiopia, Tanzania and the Philippines.
